# Increasing molecular diagnostic capacity and COVID-19 incidence in Brazil

**DOI:** 10.1017/S0950268820001818

**Published:** 2020-08-18

**Authors:** Rejane Maria Tommasini Grotto, Rodrigo Santos Lima, Gabriel Berg de Almeida, Claudia Pio Ferreira, Raul Borges Guimarães, Micheli Pronunciate, Edmur Azevedo, Rafael de Castro Catão, Carlos Magno Castelo Branco Fortaleza

**Affiliations:** 1 School of Agriculture, São Paulo State University (Unesp), Botucatu, Brazil; 2 Botucatu Medical School, São Paulo State University (Unesp), Botucatu, Brazil; 3 Clinical Hospital of Botucatu Medical School (HCFMB), Botucatu, Brazil; 4 Institute of Biosciences, São Paulo State University (Unesp), Botucatu, Brazil; 5 School of Technology and Sciences, São Paulo State University (Unesp), Presidente Prudente, Brazil; 6 Federal University of Espirito Santo (UFES), Vitória, Brazil

**Keywords:** COVID-19, diagnosis, epidemiology, infectious disease, laboratory tests

## Abstract

Different countries have adopted strategies for the early detection of SARS-CoV-2 since the declaration of community transmission by the World Health Organization (WHO) and timely diagnosis has been considered one of the major obstacles for surveillance and healthcare. Here, we report the increase of the number of laboratories to COVID-19 diagnosis in Brazil. Our results demonstrate an increase and decentralisation of certified laboratories, which does not match the much higher increase in the number of COVID-19 cases. Also, it becomes clear that laboratories are irregularly distributed over the country, with a concentration in the most developed state, São Paulo.

Different countries have adopted strategies for the early detection of SARS-CoV-2 since the declaration of community transmission of the virus by the World Health Organization (WHO), allowing early clinical intervention and the management of these patients with non-pharmacological measures such as hospital isolation, suspension of regular activities and respiratory support in intensive care units (ICUs) promptly after diagnosis [1]. Timely diagnosis has been considered one of the major obstacles for surveillance and healthcare (especially hospital) preparedness in low-to-middle income countries [2]. With that in mind, we studied the increase in COVID-19 molecular diagnostic capacity of public health laboratories in different regions in Brazil. We were especially interested in analysing the association of newly certified laboratories with the increase of COVID-19.

Therefore, we searched the epidemiological bulletins provided by the Ministry of Health of Brazil (available at https://covid.saude.gov.br/) for the weekly incidence of laboratory-confirmed cases; and the Union Official Diary (a daily publication of the Federal Government official decrees, available at https://www.jusbrasil.com.br/diarios/DOU/) to identify new certifications of public laboratories for the molecular diagnosis of SARS-CoV-2. Our analysis was carried out until 4th June, when the introduction of SARS-CoV-2 in the country completed 100 days. Both the number of newly certified laboratories and the weekly incidence of laboratory-confirmed COVID-19 were submitted to Joinpoint Regression, using software Joinpoint 4.8 (National Cancer Institute, Calverton, MD) [3]. This analysis detects changes in rate trends, and was performed using a log link function to fit the data. We also performed univariate and single-step multivariable Poisson Regression model, with the number of laboratories and the epidemiological weeks as predictors for the outcome of interest (rate of COVID-19 confirmed cases), using STATA 14 (Statacorp, College Station, TX), and georeferenced the time of introduction of COVID-19 and certification of laboratories in different areas in Brazil, using ArcGIS 10 (ESRI, Redlands, CA). We then applied the inverse distance weighted (IDW) technique to interpolate discrete cases and transform it in a continuous surface in raster format, highlighting the date of case arrival and the geographic region. IDW is a local deterministic interpolator which does not exceed data intervals between neighbours. We used 20 neighbours and a 1.5 power factor. The diffusion layer was overlaid with the laboratories mapped by municipality and week of certification.

Our results are shown in Figure 1. We can observe on the map (panel C) the COVID-19 spatial diffusion pattern, starting in the main metropolis throughout the country, mainly São Paulo, Rio de Janeiro, Fortaleza, Recife and Manaus. The diffusion follows the path of the urban network going from major metropolis to middle size cities and then to small cities. The velocity of the spread is different among the regions of the country. In North of Brazil, the diffusion was extremely fast despite lack of road transport in several portions. In Northeast and Southeast regions, metropolitan areas and cities with higher populational density concentrated the early cases of COVID-19, spreading after towards the interior of each region. In South and Midwest regions, the diffusion was at the initial stages, centred in major urban areas and near major road axis.

**Fig. 1.**
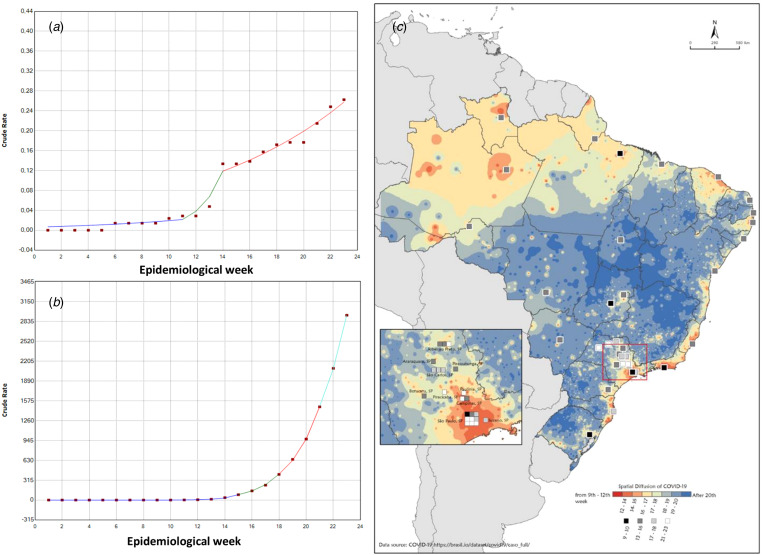
Trends on SARS-CoV-2 infection and laboratory capacity in Brazil over one hundred days since first COVID-19 confirmed case. Section A shows the rate of certifications of public laboratories for the molecular diagnosis of SARS-CoV-2 by epidemiological week, in a Joinpoint Regression analyzes. Section B shows the rate of incidence of laboratory-confirmed cases by epidemiological week, also in a Joinpoint Regression analyzes. Section C shows temporal-spatial diffusion of COVID-19 in Brazil: warm colors designate early introduction (i.e. 12–14 epidemiological weeks), while cool colors designate recent introduction (i.e. 19–20 epidemiological week). Certified laboratories for SARS-CoV-2 infection molecular diagnosis are represented by squares in greyscale also in section C. The shades of grey range from the darkest for the three initial certified public health laboratories to the light grey representing those that were certified in later stages of the outbreak.

Panel A shows the rate of certifications of public laboratories for the molecular diagnosis of SARS-CoV-2 by epidemiological week, whereas panel B shows the rate of incidence of laboratory-confirmed COVID-19 by epidemiological week, both in a Joinpoint Regression analysis (per 100 000 inhabitants). One can note an increase of certified laboratories, which does not match the much higher increase in the number of cases.

The laboratory's certification ranged from few laboratories (one in the North-Northeast region, one in the Middle East and South and two in Southeast) to 26 labs, in 8 weeks. Almost all regional centres of the country certificated at least one laboratory. In summary, at week 23 the ratio between the number of laboratories to population density (population per km^2^ at the last census at 2010) at each Brazil's region was 6:4.14, 4:8.75, 28:86.92, 3:48.58 and 7:34.15, respectively, at North, Middle East, Southeast, South and Northeast. It is also clear from panel C that laboratories are irregularly distributed over the country, with a concentration in the most developed state, São Paulo (13 of 35 laboratories). However, a decentralisation trend can be seen over the last epidemiological weeks, highlighted by the presence of newly certified laboratories specially in North and Northern regions of Brazil. It is important to reveal that in São Paulo State there are many cities performing mass testing, and this could explain the greater growth in the number of labs. Interestingly, the number of laboratories is positively associated with the number of COVID-19 cases in the univariate model (incidence rate ratio (IRR), 1.11; 95% confidence interval (CI), 1.11–1.11) but negatively associated after adjusting for epidemiological week (number of laboratories: IRR, 0.98; 95% CI 0.97–0.99; epidemiological week: IRR, 1.70; 95% CI 1.69–1.71). This finding can be interpreted in two directions. From an optimistic perspective, the slow increase in certifications of new laboratories for diagnosis does not necessarily correlates with the overall diagnostic capacity of the laboratory net, once already certified individual laboratories may increase their own capacity as well. This analysis could not be performed as no data were available regarding diagnostic capacity of each one of these laboratories. From a pessimistic perspective, the fast increase of COVID-19 incidence and the continuous spread into inner country, less developed areas of Brazil challenges diagnostic capacity and therefore, accurate and timely health surveillance [4]. This implies that, given the fast increase of COVID-19 cases and the continuous spread into inner Brazil [5], the laboratories (which are continuously increasing their capacity) may still be insufficient to provide accurate data in a setting of exhaustion of hospital (especially ICUs) capacity [6]. The primacy of the growth of cases over laboratory capacity is reinforced by the increase in hospital admissions and deaths (as reported in official data (https://covid.saude.gov.br/) and recent studies [7, 8]).

Health surveillance has been a strong pillar of response to previous public health emergencies in Brazil, including pandemic H1N1 influenza and Zika virus [9]. Challenges for COVID-19 response are not restricted to health surveillance [10], but strengthening an accurate knowledge of its behaviour can direct preventive strategies (including infection control). Serial antibody prevalence surveys may be an option but are still hampered by inaccurate serological tests [11]. Given that quarantine measures started to be relaxed in national territory, this is a critical moment where diagnosis missing can jeopardise the epidemic control. Therefore, Brazilian states must increase their capacity of timely molecular diagnosis, not only to face this pandemic, but as a network for preparedness for future public health emergencies [12].

## Data Availability

The authors state that the database used in the analyses can be available as a supplementary file to the paper or provided to interested researchers upon reasonable request.
